# Diatom-Positive Cadaver: Drowning or Homicide?

**DOI:** 10.7759/cureus.18312

**Published:** 2021-09-27

**Authors:** Aiman Khurshid, Mir U Shah, Maman Khurshid, Aruba Sohail, Gulzar Ali

**Affiliations:** 1 Forensic Medicine, Civil Hospital Karachi, Karachi, PAK; 2 Internal Medicine, Dow University of Health Sciences, Karachi, PAK; 3 Forensic Medicine, Dow University of Health Sciences, Karachi, PAK

**Keywords:** homicide, forensic medicine, diatoms, autopsy, exhumation

## Abstract

Medico-legal investigations should be performed on all unnatural (homicide, suicide, or accident), unexpected, and suspicious deaths to evaluate the possibility of homicide and ascertain the exact cause of death. However, in some scenarios, burial takes place before an autopsy can be conducted. In such cases, exhumation is performed, which involves excavating the remains of previously buried or cremated individuals for medico-legal investigations. Although the diatom test is a very useful microbiological approach in concluding death by drowning, its reliability remains controversial. Our study presents the case of a cadaver that was exhumed so that medico-legal investigations could be performed to ascertain the exact cause of death. The cadaver was recovered from water but buried before an autopsy could be performed. Upon exhumation, the greater cornu of hyoid bone was fractured with dislocation of the maxilla and mandible. The femur, sternum and clavicle were sent for diatom testing. The diatoms came out positive in the bones; however, the water sample from the gutter didn’t test positive for diatoms. Thus, due to the diatom-negative status of water, diatoms from bones can’t be compared with suspected water samples. Since diatoms in bones can arise as a result of contamination too, death cannot be concluded by drowning. Manual strangulation led to the fracture of the hyoid bone. Asphyxia due to throttling was declared the cause of death. Due to the unreliability of the diatom test in certain cases, other tests should be performed in auxiliary to the diatom test to conclude death by drowning.

## Introduction

Medico-legal autopsies are performed as per the laws of each country, and the findings are admissible evidence in the court of law. All unnatural (homicide, suicide, or accident), unexpected, and suspicious deaths should undergo a medico-legal investigation to evaluate the possibility of homicide and ascertain the exact cause of death. However, in some scenarios, burial takes place before an autopsy can be conducted. In such cases, exhumation is performed, which involves excavating the remains of previously buried or cremated individuals for medico-legal investigations, identification, relocation, or other purposes.

When dealing with a body recovered from the water, it remains challenging to identify the cause of death, especially distinguishing drowning from other causes of death [1]. If a certain number of diatoms are found in the victim’s organs, the cause of death is believed to be drowning, and the presence of diatoms has been considered to be the “gold standard” for drowning identification [2,3]. Although it is the most widely accepted method for identifying drowning, there are major discrepancies in the validity and reliability of diatom testing in the diagnosis of drowning [4]. The use of diatom testing to diagnose drowning has received much criticism since it was first introduced, and it has not proven useful in the majority of forensic drowning cases [5]. In studies conducted in the past, diatoms were detected in only 1/3rd of fresh-water drownings [6-8]. Meanwhile, some authors pointed out that it is not impossible for diatoms to enter other organs from the intestine, which could give false positives and obscure the actual cause of death [9]. Moreover, false positives could also be obtained as a result of contamination [10]. Thus, a body recovered from water doesn’t necessarily imply that the death was due to drowning alone. A dead body in the water could be a result of disposal after death. Also, there is a possibility that death is a consequence of both drowning and homicide [7]. Therefore, it is essential to conduct medico-legal investigations of a body recovered from water to rule out any possibility of a homicide.

In this case report, we describe a cadaver that was exhumed so that medico-legal investigations could be performed to ascertain the exact cause of death. The cadaver was recovered from water but buried before an autopsy could be performed. 

## Case presentation

A dead body of a 34-year-old male was found from a gutter line in Thatta district, Sindh. Upon external examination, the dead body was found to have widespread blisters and swollen face and abdomen. The macerated foul-smelling body with a half-opened mouth was missing nose, upper lip, and left eyelid as a result of being eaten by fish/insects dwelling in the gutter. The clothes, hair, nails, and nail scrapings of the dead body were sent for DNA and serological analyses. Upon serological analyses, blood was detected on the clothes of the victim. The DNA profile obtained from the hair and nail of the victim was consistent with a single source of male origin. The DNA profile obtained from nail scrapings was a mixture of at least two individuals with a major and minor component. The major component matched the DNA profile of the hair and nail of the deceased, but the minor component did not match, which further adds weight to the possibility of a homicide. The dead body was then buried without forensic intervention due to a lack of forensic expertise in the rural area of Thatta.

**Figure 1:**
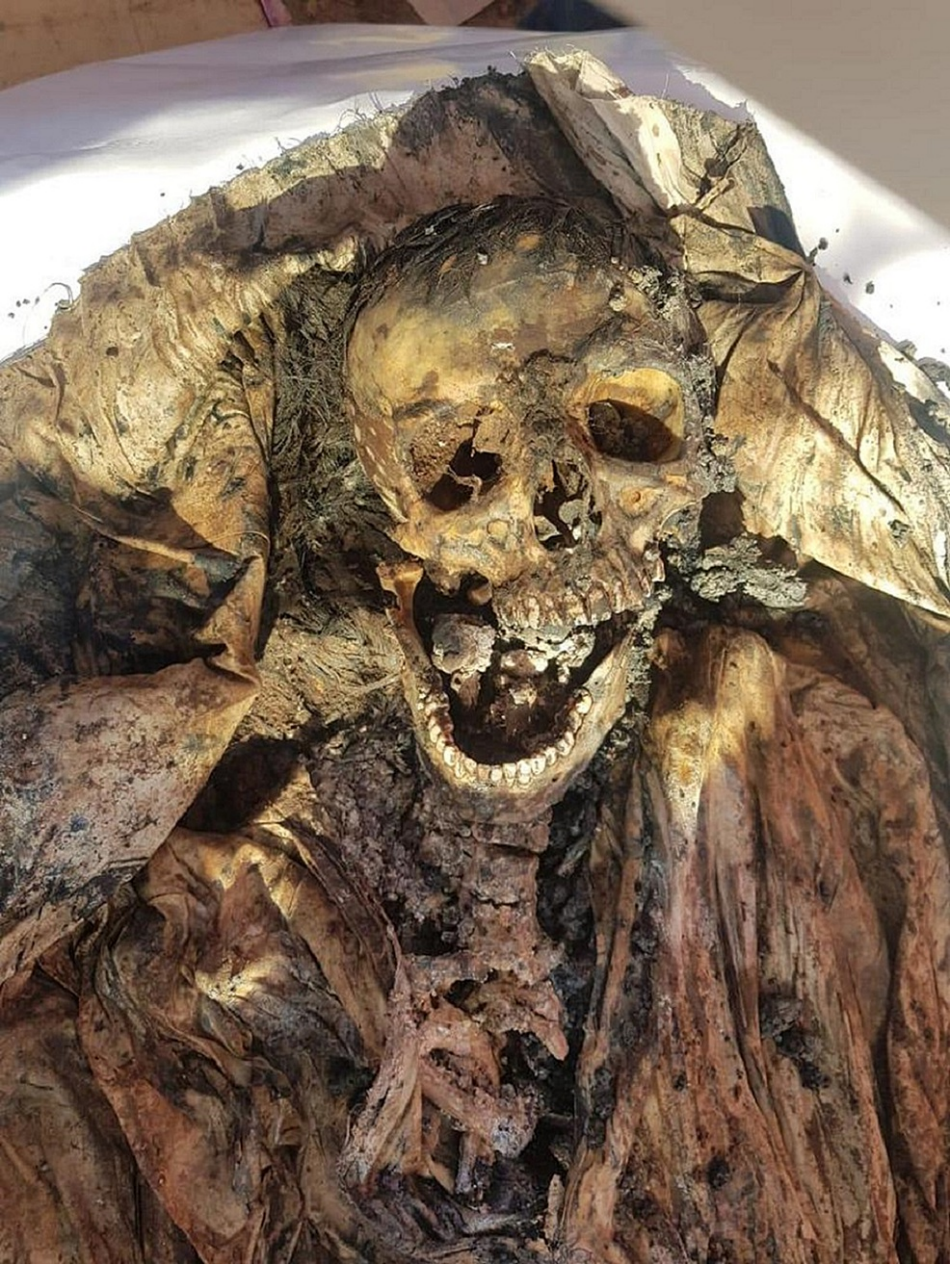
Skull and neck of the exhumed body showing an advanced stage of decomposition.

**Figure 2:**
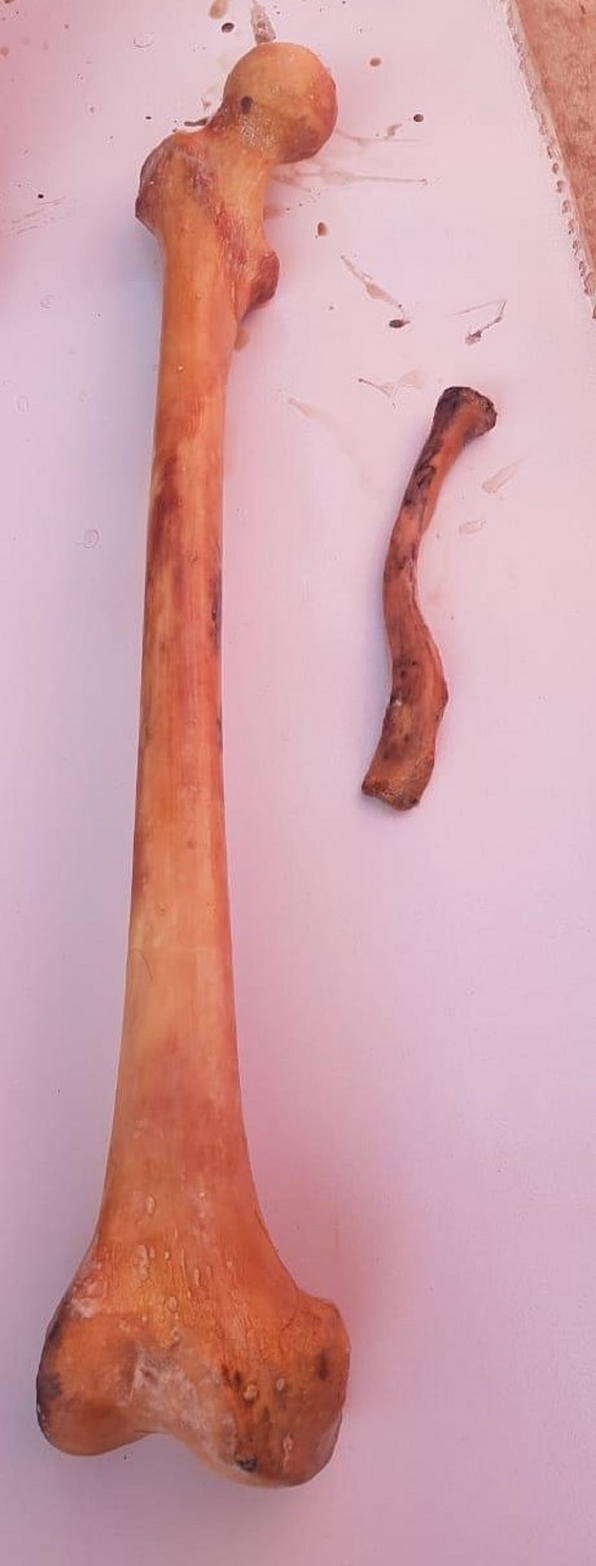
Right femur (left) and left clavicle (right).

After almost six months post burial, exhumation of the dead body was requested by the investigating officer to ascertain the cause of death, whereafter, it was performed. Upon unearthing, the body was found in an advanced stage of decomposition, with soft tissues decomposed and the underlying skeleton intact (Figure 1). Greater cornu of right upper 1/3^rd^ of hyoid bone was found fractured, and both maxilla and mandible were dislocated (Figure 1). The rest of the skeleton was unremarkable in appearance. The left clavicle and right femur were reserved for chemical analysis to detect the presence of diatoms and traces of poison (Figure 2). Water from the death site (gutter) was taken to compare and detect diatoms. The right clavicle bone and two pairs of teeth were also extracted for parental testing. The cause of death was reserved till results from the chemical analysis were received.

The DNA matched with the blood samples of the parents, which confirmed the identity of the dead body. The chemical analysis excluded poisoning and confirmed diatoms’ presence in the reserved bones. However, diatoms were not found in the water sample from the death site. Fracture of the greater cornu of the right upper 1/3rd of hyoid bone suggested manual strangulation. From the aforementioned findings, asphyxia, as a result of throttling, was declared the cause of death.

## Discussion

Although the recovery of the cadaver from water was highly suggestive of drowning, medico-legal investigations confirmed the occurrence of a homicide. As the minor component of the DNA profile obtained from nail scrapings didn’t match the cadaver’s DNA, its possible source could be the suspect responsible for the homicide. Violent crimes are often associated with intense physical contact between the victim and the perpetrator due to struggling at the scene of the crime. This facilitates the transference of blood, epithelial cells, semen, or any other biological material between the two individuals, which could collect under the suspect's or the victim’s fingernails [11,12]. Thus, finding foreign biological material under the fingernails of the cadaver can be valuable evidence in the investigation of a homicide. 

In this case, diatom findings have been inconclusive in determining drowning to be the cause of death. Although diatoms were found in the bones reserved for chemical analysis, the sample of water taken from the drowning site tested negative for diatoms. This could be a result of improper preservation of water samples from the gutter line. Furthermore, due to the “diatom-negative" status of water, diatom species abundance patterns from bones can't be compared with the suspected water samples. Thus, the source of diatoms in the bones cannot be concluded to be the suspected drowning site. Additionally, diatom positivity in bones does not rule out the possibility of homicide since false-positive results are common in diatom testing, as can be seen in bodies dumped in water post homicide. False positives can arise as a result of contamination of laboratory procedures and intake of diatoms in food when alive [13]. Furthermore, some authors have suggested the possibility of false-positive diatoms' results due to ante-mortem penetration of diatoms in the bloodstream through the intestinal or respiratory tract [4]. As false positives can occur due to the aforementioned reasons, it is necessary to compare the diatoms from the bones with that of suspected water samples to establish a drowning diagnosis. However, the inability to compare the diatom species abundance patterns in our case limits the efficacy of the diatom test to conclude death by drowning.

Upon exhumation, greater cornu of right upper 1/3^rd^ of hyoid bone was found to be fractured, which is suggestive of strangulation. In an experimental study simulating manual strangulation, Lebreton-Chakour et al. found that a majority of the fractures were observed in the hyoid bone at the synchondrosis or on the greater horns [14]. During the external examination conducted before the initial burial, no ligature mark was observed, which is highly suggestive of manual strangulation. In other studies, 68.3% [15], 70% [16], and 51% [17], of manual strangulation cases had laryngohyoid fractures, more often in connection with a manual than with ligature strangulation [17]. Thus, asphyxia as a result of manual strangulation was the most likely conclusion in this case. 

As diatom tests are not always conclusive in drowning diagnosis, other tests should also be used in conjunction with diatom tests. Polymerase chain reaction (PCR) detection for green algae and cyanobacteria can serve as an auxiliary for drowning diagnosis, as they can be positive in drowning cases where diatom testing was negative [10]. New methods in diatom testing are increasingly being developed worldwide. The microwave digestion-vacuum-filtration-automated scanning electron microscopy (MD-VF-Auto SEM) method and the addition of deep learning will make diatom testing even more effective in drowning diagnosis [10]. These methods could increase the reliability of diatom tests and prevent false-positive results; hence they should be implemented in Pakistan as well.

## Conclusions

In certain cases, dead bodies are buried before any medico-legal investigation can be conducted due to religious reasons or lack of forensic expertise. If an autopsy is not performed before burial, exhumation can be conducted, which involves excavating the remains of previously buried or cremated individuals. Our case report demonstrates that it is essential for all unnatural (homicide, suicide, or accident), unexpected, and suspicious deaths to undergo a medico-legal investigation to exclude foul play. Upon exhumation, experts found greater cornu of the right upper 1/3^rd^ of the hyoid bone to be fractured, which was suggestive of manual strangulation. Additionally, the diatom test was inconclusive to confirm death by drowning. Thus, other tests such as PCR detection for green algae and cyanobacteria should be performed in addition to the diatom test to confirm a drowning diagnosis.
